# Neuron matters: electric activation of neuronal tissue is dependent on the interaction between the neuron and the electric field

**DOI:** 10.1186/s12984-015-0061-1

**Published:** 2015-08-12

**Authors:** Hui Ye, Amanda Steiger

**Affiliations:** Department of Biology, Loyola University Chicago, 1032 W. Sheridan Rd, Chicago, IL 60660 USA

**Keywords:** Electric stimulation, Tissue properties, Cell-field interaction, Modeling, Deep brain stimulation (DBS), Transcranial direct current stimulation (tDCS), Transcranial magnetic stimulation (TMS)

## Abstract

In laboratory research and clinical practice, externally-applied electric fields have been widely used to control neuronal activity. It is generally accepted that neuronal excitability is controlled by electric current that depolarizes or hyperpolarizes the excitable cell membrane. What determines the amount of polarization? Research on the mechanisms of electric stimulation focus on the optimal control of the field properties (frequency, amplitude, and direction of the electric currents) to improve stimulation outcomes. Emerging evidence from modeling and experimental studies support the existence of interactions between the targeted neurons and the externally-applied electric fields. With cell-field interaction, we suggest a two-way process. When a neuron is positioned inside an electric field, the electric field will induce a change in the resting membrane potential by superimposing an electrically-induced transmembrane potential (ITP). At the same time, the electric field can be perturbed and re-distributed by the cell. This cell-field interaction may play a significant role in the overall effects of stimulation. The redistributed field can cause secondary effects to neighboring cells by altering their geometrical pattern and amount of membrane polarization. Neurons excited by the externally-applied electric field can also affect neighboring cells by ephaptic interaction. Both aspects of the cell-field interaction depend on the biophysical properties of the neuronal tissue, including geometric (i.e., size, shape, orientation to the field) and electric (i.e., conductivity and dielectricity) attributes of the cells. The biophysical basis of the cell-field interaction can be explained by the electromagnetism theory. Further experimental and simulation studies on electric stimulation of neuronal tissue should consider the prospect of a cell-field interaction, and a better understanding of tissue inhomogeneity and anisotropy is needed to fully appreciate the neural basis of cell-field interaction as well as the biological effects of electric stimulation.

## Introduction

As an alternative and supplementary treatment strategy to pharmacological approaches, electrical stimulation has been widely used for the treatment of many neurological disorders. In such, deep brain stimulation (DBS) with inserted electrodes is rendered effective for the treatment of Parkinson’s, essential tremor, and dystonia [1]. Transcranial direct current stimulation (tDCS) applies non-invasive, painless brain stimulation with large electrodes attached to the scalp, which provide beneficial effects in neurological disorders such as Alzheimer’s disease [2] and Parkinson’s disease [3]. It also improves motor function after stroke [4]. Alternating currents applied via transcranial alternating current stimulation (tACS) over the occipital cortex of the brain can entrain neural oscillations of the underlying brain [5]. In laboratory research, direct current (DC) electric stimulation has been shown to suppress *in vitro* seizure activity [6, 7]. High intensity electric fields have been used to increase permeability of the cell membrane [8, 9] for efficient delivery of medicine and genes to the target tissue [10].

Aside from direct application via stimulating electrodes, electric current can also be delivered by electromagnetic induction with time-varying magnetic field inside the biological tissue, activating the cells and providing non-invasive stimulation [11]. The effects of magnetic stimulation on excitable biological tissues have been experimentally studied since the start of the 20^th^ century through the pioneering works of Jacques d’Arsonval (1896) and Silvanus P. Thompson (1910) in human visual sensations, i.e., magnetophosphenes. In 1982, Polson and Barker applied the first successful stimulation of peripheral nerves through the use of magnetic fields [12]. In 1985, Barker performed the first non-invasive magnetic stimulation of human motor cortex [13]. Today, transcranial magnetic stimulation (TMS) is explored in the treatment of depression [14], epilepsy [15] and Parkinson’s disease [16]. Magnetic field has also been used to control muscle spasticity [17], and in the diagnosing and charting of disease and mechanical damage in peripheral nerve pathways.

Effects of an electric field on neuronal tissue are caused by an establishment of a transmembrane potential. When a neuron is positioned inside an electric field, the electric field will induce a change in the resting transmembrane potential by superimposing an electrically-induced transmembrane potential (ITP). When the electric current penetrates the membrane, the neuronal membrane may be depolarized and/or hyperpolarized from its resting value (i.e., −70 mV), which causes excitation or inhibition of the cell. The depolarization may reach the threshold to generate an action potential, which involves the opening of voltage-gated ion channels such as sodium and potassium channels [18]. Under extreme conditions, a large induced potential can generate a configuration change in the membrane, forming pores and instigating a phenomenon known as electroporation [19].

In this review, we focus our questions on 1. How is ITP measured and calculated during electric or magnetic stimulation? 2. What might be the impact factors on the establishment of the ITP across the neuronal membrane? We show that interactions between the targeted neurons and the externally-applied electric fields play significant roles in generating ITP. We argue that a better understanding of tissue inhomogeneity and anisotropy is crucial for the full appreciation of neural basis of cell-field interaction, as well as the biological effects of electric stimulation.

## Review

### Computation and measurement of the induced transmembrane potential (ITP) during electric and magnetic stimulation

Both modeling and experimental approaches have been used to estimate the ITP during electric stimulation. The mathematical basis of modeling work is to solve Maxwell equations [20], which describe the electric potential distribution in three-dimensional space. Analytical solutions for a single cell ITP became available as early as the 1950s [21, 22]. Later on, more complicated cellular geometry and stimulation diagrams have been investigated to analytically solve ITP [23, 24]. In the last 20 years, numerical approaches using multi-compartment models have been carried out to study ITP in biologically authentic neuronal structures under electric field stimulation (i.e., [25, 26]) by taking advantage of modern computer power. Several successful simulation software packages have been implemented to simulate the neuron and network activity under electric stimulation. One such platform is NEURON [27], a simulation environment developed at Yale and Duke (http://www.neuron.yale.edu/neuron/) capable of stimulating active ion-channel mechanisms.

In parallel to theoretical studies, direct measurements of ITP were reported ranging from hippocampal neurons with sharp electrodes [28] to neural stem cells with patch electrodes [29] with certain technical challenges arising from artificial noise introduced by the externally-applied field. A relatively easy and more reliable method is to use the voltage-sensitive dyes [30], which have shown great potential in their ability to measure ITP under electric field stimulation. Voltage-sensitive dye provides enough resolution for the polarization on the cell membrane under electric stimulation. For example, it can be used to validate the theoretical prediction of the ITP distribution on a cell under DC field stimulation [30].

Estimations on ITP during magnetic stimulation have been equally successful with a primary focus on axonal responses to the fields [31]. In example, analysis of the ITP of an axon located at the center of a nerve bundle [32] led to the successful derivation of a generalized cable equation for magnetic stimulation of an axon [33], a well-known equation that describes voltage distribution along the axon during electric stimulation. The ITPs for a spherical cell under time varying magnetic stimulation [34] and an unmyelinated axon stimulated by a magnetically-induced electric current that transverses the axon [35] have been recently solved.

Although the electric and magnetic stimulations share the same biophysical mechanisms in activating the neurons, they are not interchangeable in neither clinical nor experimental practices. At a macroscopic tissue level, the electric field further from the stimulating electrodes may be significantly dispersed by the non-homogenous tissue conductivity during electric stimulation. In contrast, the induced electric field may be less dispersed during magnetic stimulation because the magnetic field may penetrate through the tissue without significant attenuation. Therefore, with careful coil design and placement around the head, the magnetic field can achieve good stimulation in the brain region such as the motor cortex [36, 37]. At a microscopic single cell level, the geometrical patterns of ITP on the cell membrane [34] could be dramatically different from that in electric stimulation [20, 24, 38], suggesting that individual cell activation may be significantly different across these two stimulation protocols.

### Effects of field properties on neuronal polarization

What determines the amount and pattern of cellular polarization in electric stimulation? First, the properties of the externally-applied electric field play significant roles in membrane polarization; these include *direction, intensity, frequency*, and *waveform* of the field. Amplitude and frequency of the stimulation current are the key parameters in deep brain stimulation [39, 40]. Orientation of the retinal neurons to the micro-fabricated multi-electrode array determines their activation [41]. Similarly, during the electrical stimulation of cardiac cells, the threshold for excitation depends on the orientation of the electric field to the target cells [42, 43]. Cellular response to stimulation could be frequency-dependent due to the non-linear biophysical properties of the cell membrane. High frequency electric field proves more efficient in cell penetration and subsequent effect on internal organelles such as mitochondria than lower frequency electric fields [23, 44].

Similarly, the characteristics of the magnetic field have been tightly related to the effects of stimulation as they define the properties of the induced electric current in the tissue. Among these parameters, three stand out and dictate the bulk of the overall outcomes. The *frequency* of the externally-applied magnetic field determines the strength of the induced field by the law of electromagnetic induction [45]. The *magnitude* of the magnetic field determines the intensity of the induced electric field. Both these quantities, in turn, affect the extent of polarization in a single cell [34]. Lastly, the *orientation* of the magnetic field in relationship to the neurons determines the direction of the induced electric field and the geometric pattern of polarization in the neuronal tissue [34, 46]. Consequentially, neurons in the motor cortex displayed different sensitivities to transcranial magnetic fields under stimulation with differing coil orientations and shape of the induced current pulse and intensity [36, 37, 47–50].

**Fig. 1:**
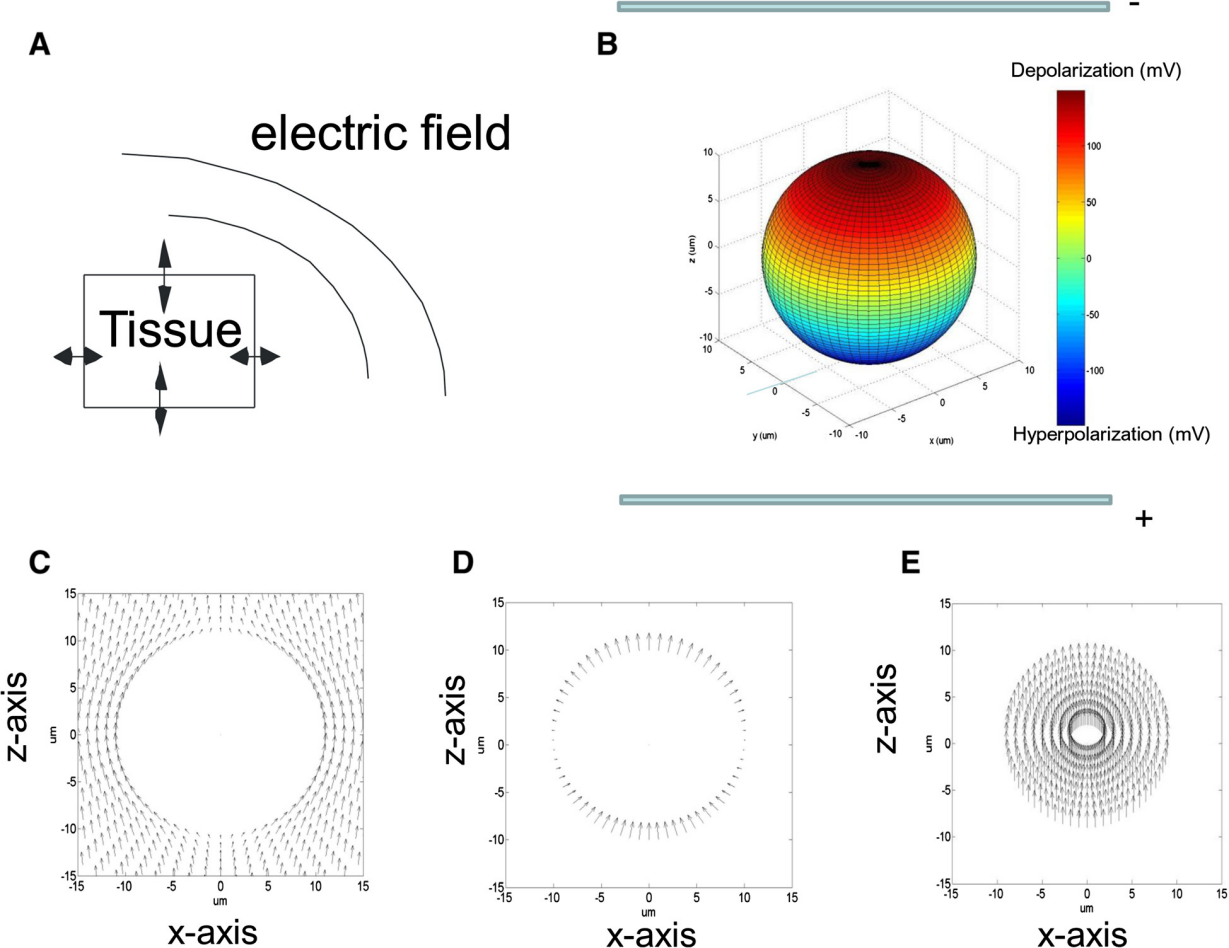
**a**. Conceptive illustration of the interaction between the biological tissue and the electric field which determines the overall polarization of the tissue. This interaction caused both cellular polarization and redistribution of the externally-applied field. **b**. Polarization pattern of a 3D model cell in a DC electric field generated by a pair of parallel-plate electrodes. The color bar indicates the amount of membrane potential change due to electric stimulation. All the parameters for this modeled cell is listed in [74]. **c**. Extracellular electric field distribution on the x-z plane (y = 0) (viewing the sphere from the y-axis). **d**. Electric field inside the membrane; **e**. Intracellular field distribution. Figures **c** to **e** are adapted from [74], with arrows to represent the direction of the electric fields. The size of the arrows represents the intensity of the electric field in each plots. For illustration purposes, size of the arrows are not in the same scale in **c**, **d**, and **e**. The maximal field intensity is 2531.6 V/m in **c**, 2.98 X 10^7^ V/m in **d** and 74.5 V/m in **e**, respectively

Evidence presented thus far suggests a one-way process in electric stimulation of neuronal tissues, i.e. the cell membrane is passively polarized by the external field. We, however, hereby argue that a two-way interactive process exists between the neuron and the field: the cell - field interaction. We agree that the cell is directly polarized by the external field, but we propose that the electric field is perturbed and re-distributed by the neuronal tissue (Fig. 1). The following sections will review the second aspect of the cell-field interaction and its implication in experimental and theoretical studies on electric stimulation of neuronal tissue.

### Evidence of the counter-effects of the neuronal tissue on the electric field

We first define the electric field around a cell. An electric field around a single cell is separated into three major areas: the field in the extracellular medium (extracellular field), the field within the membrane (transmembrane electric field), and the field in the cytoplasm (intracellular field).

Electric fields in these distinct regions all possess important physiological significance. Extracellular electric field has been recorded and analyzed in the study of cellular communication and synchronized excitability. Direct measurement and computation of this field has been a focus for research interested in electric stimulation on neuronal tissue. Equally important, the transmembrane electric field maintains the meta-stable membrane structure [51], and is essential for the manipulation of the transmembrane potential during electroporation [52] and tumor electrochemotherapy [10, 53]. Finally, when an electric field penetrates into the cytoplasmic space (intracellular field), it instigates the polarization of internal organelles [44, 54] and facilitates polynucleotide uptake and migration within the cell [55].

Initial evidence of the counter-effect of the neuron to the externally-applied field is seen in the significant disruption and redistribution of the local field when the field is sensed by the cell in question. Models with both analytical and numerical methods support this notion. For example, in an evenly distributed DC field, the electric field could be greatly augmented inside the cell membrane [56]. Jerry et al. have presented that electric fields inside and outside of a cell are, both, affected by the shape of the cell [57]. Numerical solutions indicate that the presence of a spherical cell inside a field could perturb the electrical potential generated by a microelectrode [30]. A strong electric field will generate pores on the cell membrane in electroporation, and presence of these pores alter the electric field distribution inside and outside the cells [58].

A direct consequence of the counter-effects of the cell to the electric field is that electric field alteration by one individual cell may cause secondary effects on neighboring cells during electric stimulation, particularly in the scenario that two or more cells are located in close proximity and in a high-density cell medium. Evidence demonstrated that the presence of a single cell affects ITP in its neighboring cells without direct physical contact between the two cells [59]. Though single cell ITP follows a predictable pattern of distribution under a specific stimulation electrode arrangement [30], the polarization patterns are fairly complex in a large cluster cell population, suggesting an interactive effect among neighboring cells. This complexity is more apparent for central cells than those at the periphery [60]. In majority of experiments that cells are exposed to the electric filed, cells are surrounded by other cells. It was found that the ITP in individual cells depends on the overall density and spatial arrangement of the cells in the three-dimensional cell cluster [8]. A reduction in cell density would potentially decrease cellular interaction via electric field coupling.

### Cell communication via ephaptic interaction

Since an externally-applied electric field can activate neurons and the presence of neurons can alter extracellular electric field distribution, it is likely that neurons excited by the externally-applied electric field can also affect neighboring cells via ephaptic interaction. The functional significance of direct electric (ephaptic) interaction was largely ignored until the 1960s when an inhibitory action on Mauthner cells in goldfish was discovered [61]. A single impulse in the Mauthner cells is capable of causing nearly simultaneous firing of 40–80 interneurons [62]. In another example, the isthmo-optic cells in pigeons can be excited by an electrical field effect (ephaptic interaction) that is produced by the nearby cells whose axons were activated, which suggests that electrical field effect may play important roles in interneuronal communication [63]. Moreover, it was suggested that cells can communicate via electrical interactions mediated across extracellular space, and that field effects could facilitate synchronized and even epileptic-like neuronal bursting [64] and neuron-glia communication [65]. External electric stimulation via the interruption of ephatic interaction between neurons has been proposed as a key neuronal mechanism for use in seizure suppression [66].

### Role of tissue properties on cell-field interaction

What may affect the cell-field interaction during electric stimulation? In the following sections we will present evidence that both aspects of the interaction (the ITP and the re-distributed electric field around the polarized cell) depend on the inhomogeneous properties of the target tissue.

At a microscopic level, cell property falls under two categories of measurement: the geometric and the electric parameters of the cell. Geometrical properties include the shape and size of the cell, as well as its orientation and location in the field. Electrical properties include tissue conductivity and dielectricity in the microscopic cellular environment, including the properties of the extracellular media, the cell membrane, and the internal cellular environment.

### Effect of tissue properties on cell polarization

Shape of the target neurons plays a significant role in determining the amplitude and pattern of distribution of ITP during electric and magnetic stimulations. By comparing different ITPs in a spherical cell [20] versus a spheroidal cell [38] under the same electric field, we can see that ITP pattern in a spheroidal is much more complicated (and harder to compute) than that in a spherical cell.

Cell radius is also important in determining the amplitude of the ITP [20]. Larger cells are associated with greater ITPs [34] and require lower external fields to create permeable cell membranes [19]. In DBS, it is established that relative magnitude of the effects within different brain regions is dependent on axon fiber size [67]. Similarly, electroporation studies have demonstrated that the threshold for membrane permeabilization is associated with the size of the target cells [68, 69].

Orientation of the cell to the externally-applied field determines both the amplitude and pattern of ITP within the cell [20, 24]. For a point electrode, ITP is dependent on the electrode-to-cell distance [30]. The threshold for excitation of the retina ganglion cell axons is dependent on the orientation of the electric field to the axons [41]. At gross tissue level, orientation of the electrodes plays a significant role in determining the outcome of tumor electrochemotherapy [53]. Likewise, orientation of the magnetic coil is a major concern in effective transcranial magnetic stimulation of the motor cortex [37, 47, 50].

In addition to the geometrical properties of the cell, growing evidence supports that electric properties of the cellular environment affect ITP. For example, ITP induced by a microelectrode on a spherical cell is dependent on extracellular and intracellular media conductance [30]. In a spheroidal cell model, Kotnic and Miklavcic [24] revealed that ITP is a function of membrane conductivity. In the area of electroporation using extremely large ITP, both experimental [60, 70] and modeling results [71] have shown that intracellular molecular concentrations could affect pore formation. This is because large influxes of molecules through the pores will induce alteration of the ITP and, therefore, the pore configuration—affecting overall conductivity. Theoretical work has also argued the significance of medium conductivity in its effect on membrane permeability under electroporation [20, 72, 73], although experimental studies have yet to provide backing of these model outcomes.

#### Effect of tissue properties on field re-distribution

The other half of the cell-field interaction, redistribution of the field by the tissue, is also largely reliant on the properties of the tissue.

The geometrical property of the cell causes external field redistribution. Numerical modeling results [73] have shown that membrane conductivity affects the field distribution around and within an intact singular cell. The intracellular field increases greatly as membrane resistance decreases, and is dependent on cell radius and membrane thickness [74]. During electroporation, transit pore formation can change the direction of the field distribution and electric driving force near to the pore, thus, the magnitude of the electric field proximal to the poles is significantly decreased [72, 73, 75]. Therefore, alteration in cellular structure can change the field distribution around the cell.

Field distribution in the neuronal tissue also depends heavily on the electrical properties of the target tissue under electric stimulation. At a single cell level, membrane conductance affects the field distribution around and within a single cell during electric stimulation [57, 58, 72]. We further show that intra- and extracellular conductivities of the cell affect the field distribution inside/outside the cell and within the membrane [74]. In clinical practice, when treating Parkinsonian symptoms with electric stimulation of the subthalamic nuclecus (STN), increasing STN conductivity may provide better spread of the field outside of the target region [67]. In tDCS, anisotropic properties of the head play significant roles in the distribution of the electric fields and the effects of the stimulation [76, 77]. Magnetic stimulation studies also provide support that tissue heterogeneity and anisotropy can significantly alter the electric field and current density distribution induced in the brain [78–80]. Krasteva [81] assessed the magnetically-induced current in peripheral nerves and emphasized that ignoring real, non-homogenous properties of the biological structure will cause errors in evaluating the induced current. Because media conductance can affect field distribution, laboratory researchers have chosen to use a low-conductive media for DNA transfer to increase viability and transfection efficiency [68].

### Biophysics and mathematical basis of cell-field interaction and its dependency on tissue properties

The existence of cell-field interaction is rooted in the fact that most biological tissue is composed of abundant non-homogenous, anisotropic components such as the cellular/axonal membrane, the internal organelles, and the extracellular medium. The electrical properties (i.e., conductivities) of the tissue vary with its geometrical location within the tissue, even at a subcellular level, causing local accumulation of induced electric charges [82] that affect electric field distribution and ITP. From a single cell perspective, electrical charges can accumulate on the two interfaces that define the inner and outer sides of the cell membrane, also known as the extracellular medium/outer membrane interface and the inner membrane/cytoplasm interface.

As charge accumulation is a process that depends on microscopic cellular properties across the interface of two media, ITP and field re-distribution around a cell depend on both the geometrical and electrical properties of the neuron. Many works, including ours, have focused on understanding the neuronal excitation by electromagnetic field with low frequency (including DC). Under electric stimulation, the electric field distributions can be calculated by $\overset{\rightharpoonup }{\mathrm{E}}=-\nabla \mathrm{V}$. V is the electric scalar potential, due to charges and dipoles that appear as a result of application of electromagnetic field. For low frequency stimulation, we used quasi-static approximations. V can be calculated by noting that V must satisfy Laplace equation ∇^2^V = 0 in charge-free regions [23, 24]. Of course, V = 0 if charge is zero everywhere based on Coulomb’s law [45], which calculates the electric field of source charge. Consequentially, there will be no induced membrane potential (calculated as the potential difference across the membrane). In reality, presence of the surface charges, due to tissue non-homogeneity and anisotropic properties, ensures the non-zero solution electric field around the cell and ITP [20, 23].

In magnetic stimulation, two sources contribute to the magnetically-induced transmembrane electric field [82]: $\overset{\rightharpoonup }{E}=-\frac{\partial_{{}_{\mathrm{A}}^{\to }}}{\partial \mathrm{t}}-\nabla \mathrm{V}$. Here ${}_{\overrightarrow{\mathrm{A}}}$ is the magnetic vector potential, and V is the electric scalar potential. Mathematically, electromagnetic induction contributes to the magnetic vector potential term $\left(\overrightarrow{\mathrm{A}}\right)$, and ensures that the induced fields are sensitive to the coil geometry and orientation relative to the cell. Termed “primary component” [33], ${}_{\overrightarrow{\mathrm{A}}}$ has been the main focus in many computational works quantifying the magnetically-induced electric field in the tissue (i.e., [81]). The term that contributes to the transmembrane potential via surface charges (∇V), or named the “secondary term” in [33], is due to the build-up of charge on any surface between tissues with different conductivities in an inhomogeneous body.

In conclusion, both aspects of the cell-field interaction - the establishment of ITP and the redistribution of the electric field - are dependent on charge accumulation inside the biological tissue, which is caused by inhomogeneous cellular makeup and biophysical properties. The biophysical character of the neurons determines their own polarization/excitation during electric stimulation, via cell-field interaction.

### Implications for further experimental and modeling work

The reality that the electric field distribution is distorted on the interface of two non-homogenous materials (including biological tissue and other physical medium) has significant experimental implications. Currently, many researchers use invasive methods (patch or microelectrodes) to measure electric field distributions during electric stimulation. Unfortunately, the presence of these electrodes may further perturb the applied field in the tissue. To accurately quantify field distributions and transmembrane potential changes during electric stimulation, less invasive methods should be considered, such as voltage-sensitive dyes, which provide high temporal and spatial resolutions to measure potential changes in the single cells [30, 83, 84].

Parallel to the intensive experimental efforts, many mathematical modeling works have explored the mechanisms underlying the biological effects of electric stimulation. At least two distinguished types of approaches have been taken, and the discrepancy between these approaches has not been fully resolved.

The “biophysical modeling” approach simplifies the neuronal tissue with the use of basic geometrical shapes (such as a spherical cell in [20, 24, 22, 34]) and targets electrically homogeneous regions while assuming the neuronal tissue possesses a passive membrane (i.e., no active channels). Since this type of approach is completely dependent on solving fundamental physics equations with appropriate boundary conditions, it allows modelers to investigate important biophysical issues, such as the interaction between the cell and the electric field and its dependency on tissue non-homogeneity. However, this type of modeling is of limited use in understanding activation behavior of electrically-targeted neurons.

“Engineering modeling” relies on powerful computerized simulation tools to replicate biologically authentic neurons that possess complicated morphologies and active ion channels. Extracellular stimulation of target neurons is a three-step process (i.e., in [26, 25]). First, the electrical current distribution generated by the stimulation electrode is computed in the 3D space, usually using a finite element approach. Second, a multi-compartment model is built to represent the fine, geometrical structure of the neuron, with channel mechanisms incorporated into each component. Finally, the electrical field obtained from the first step is used to activate the membrane. The crucial step in this specific type of modeling is the reliable computation of the electric field distribution. Due to computational complexity, several assumptions must be made to simplify the process. First, the extracellular electric field is always treated equally around each model compartment. Second, the extracellular electric field is not affected by the presence of the tissue; and third, the extracellular voltage generated by the membrane current is neglected. Under these assumptions, the extracellular electric field is computed without considering the existence of the tissue, its non-homogenous properties, or its counter effect to the externally-applied electric fields.

These simplifications could potentially cause underestimation of the field generated by the electrode, and introduce potential certain inaccuracy in the modeling. Some investigators have acknowledged this problem in previous studies. Among them, Kotnik [54] suggested that the placement of a biological cell into an electric field leads to a local distortion of the cell in its vicinity, while Lee and Grill [30] further advised that this distortion is maximal when the electrode is in close proximity to the neuron. A recent numerical study [85] suggested that decreases in tissue conductivity could result in a decrease in the volume of neuronal tissue activated by an electrode during chronic implantation. Lastly, McIntyre et al. [26] considered potential errors introduced by the aforementioned assumptions in their simulations with NEURON while studying deep brain stimulation.

Future modeling approaches should encapsulate the biophysical concept of cell-field interaction. This is even more important for the modeling works focusing on the mechanisms of neural control by modern technologies, such as TMS and tDCS, in the treatment of neurological diseases.

Preliminary modeling studies that include some aspects of cell-field interaction have yielded a better description of the system and provided more acute simulation results that better represent biological authenticity. In the modeling of DBS of the subthalamic nucleus (STN) and its neighboring regions, Sotiropoulos and Steinmetz [67] included tissue inhomogeneity and anisotropy in the simulation. The model successfully validated that the tissue’s properties (i.e., degree of inhomogeneity and anisotropy) have a direct impact on stimulation effects, and that increasing STN conductivity could cause neuronal activation outside the STN. In tDCS, Wagner et al. [77] found that tissue anisotropy shapes the current flow in the head during tDCS, and that current tends to flow in directions more parallel to the white matter fiber tracts. In TMS, Opitz et al. [80] found that tissue types and fiber orientation affect the induced electric field. These works illustrate the importance of tissue inhomogeneity and anisotropy in the distribution of the electric field, which is part of the cell-field interaction. However, the authors did not address the cell activation by these redistributed electric currents. Furthermore, these models did not consider tissue non-homogeneities on a microscopic cellular level nor the perturbation of the field by the cell. Others focus on the excitation of the cells. Salvador et al. [37] emphasized the importance of cell geometrical properties in their activation during TMS. The paper used a homogeneous model in the calculation of the induced electric current, but ignored tissue inhomogeneity and anisotropy, and the redistribution of the induced electric field. In summary, several clinically-related modeling papers did consider some aspects of cell-field interaction in understanding the mechanisms of tDCS and TMS, but they have not considered both sides. Future works that combine “biophysical” and “engineering” modeling may provide deeper insights into the effects of electric stimulation.

We propose a hybrid method in modeling electric effects by combining “biophysics modeling” and “engineering modeling”. The core of the hereby proposed approach relies on the consideration of cell-field interactions and its dependency on tissue properties, as argued in this review. The following offers several insights into our approach: 1. Because ITP depends on tissue non-homogeneity, a detailed morphological description of the cell geometry and electrical properties at a microscopic cellular level should be implemented in the model to calculate the electric field. 2. ITP depends on the “instant” conductivities in the intracellular space, the membrane, and the extracellular space. In addition, activation of the membrane will change these conductivities through mechanisms such as voltage-gated channel opening and influx/efflux of ions. Therefore, an “instant” update of the tissue conductivities is necessary during simulations. 3. If more than one cell is modeled, as in a high-density cell medium, the interactions between the cells/axons through extracellular electric field coupling must also be considered, as the electric field generated by a polarized neuron may have “secondary” effects on its neighboring neurons/axons and vice versa.

## Conclusions

Several lines of converging experimental and modeling substantiations have suggested a broader frame of reference in which the interaction between the cells and the electric field might play significant roles in determining the ultimate polarization of the neuronal tissue under electric and magnetic stimulation. The inhomogeneous tissue property seems to affect this interaction and cellular polarization. The emerging methodology of combined physics modeling and engineering modeling, which takes into account the cell-field interaction, will provide a more accurate quantitative understanding of the neuronal activation by electric current. A joint effort of modeling and experimental research can further illustrate the significance of cell-field interaction and its biological effects during electric stimulation.
